# The Institutional Worker

**Published:** 1913-07-26

**Authors:** 


THE
### INSTITUTIONAL WORKER
**Being a Special Supplement to "The Hospital"**

### OUR BUREAU OF INFORMATION.

### Rules for Correspondents.

1. Every letter must be accompanied by the coupon to be cut from the back cover (inside page) of The Hospital, current issue, and must contain the name and address of the correspondent with pseudonym for publication if desired. Replies by post cannot be given, save under exceptional circumstances at the Editor's discretion.

2. Letters from Approved Homes in reply to special needs published in the Bureau should state terms and full particulars, and be sent prepaid under cover to the Editor of the Bureau with name written across coupon for identification.

3. Proprietors of Homes which have not yet been entered on the List of Approved Homes, but have spare accommodation likely to suit special needs, are invited to write for an application form for registration. The fee for registration, which includes two announcements of the Home in the Bureau and other privileges, is 10s.

4. All communications to be addressed to the Editor of The Hospital, 28 Southampton Street, Strand, London, W.C., and marked "Bureau of Information."

### INSTITUTIONAL FACTS AND FIGURES.

### How to Cheapen Fire Insurance.

Last week we referred to the fact that where special appliances are installed for extinguishing fires rebates are allowed in accordance with the nature of the installation. The following installations are all recognised :—(1) Brigade portable steam fire-engine or floating steam engine and a trained fire brigade to work the same; (2) manual fire-engine of modern construction, not less than 12 manual power, and a trained fire brigade to work the same; (3) boiler pumping-engine or stationary fire-engine of efficient power, with hydrants attached or in yard, and at least one hydrant connected therewith on each landing of the staircase or on each floor, the engine to be worked by power always available; (4) boiler pumping-engine or stationary fire-engine, as in No. 3, but without a hydrant on each landing or each floor; (5) two or more fire-plugs or hydrants in the yard and at least one hydrant on each landing of the staircase or on each floor, supplied with water from public waterworks, or elevated reservoirs, or other independent source with adequate constant supply; (6) two or more fire-plugs or hydrants, as in No. 5, but without hydrant on each landing; (7) portable chemical extincteur of not less than 5-gallon water capacity, having one extincteur to each 500 superficial square yards, and not less than one on each floor, or an extincteur of not less than 2-gallon water capacity, having two to each 500 superficial square yards, and not less than two on each floor, or extincteurs of not less than 1-gallon water capacity, having not less than six to each 500 superficial square yards, and not less than six on each floor; (8) buckets or cans always filled with water, having not less than six on each floor, and on every floor whose superficial area exceeds 500 square yards not less than three additional buckets or cans for each 250 square yards or part thereof after the first 500; (9) efficient portable fire-pumps having not less than one to each 500 superficial square yards, and not less than one to each floor, with adequate water supply. Allowances are also made in certain cases for automatic fire-alarms. Hospital and institutional secretaries will therefore do well to look up their fire insurances and note if allowance is being made for the installation in their institutions, for there are very few institutions that have not in some way or other protected themselves against an outbreak of fire. It will be thus seen that by a slight rearrangement, and possibly by overhauling the present policies, a substantial saving in the amount paid away in insurance premiums can be obtained.

QUESTIONS FOR JULY.

**No. 1. (For the Officers of all Medical Institutions.)—State the character of Institution, and describe your system of recording the admission, treatment, and discharge of patients admitted to the wards. Give descriptions of the various forms in use for the purpose, the method of dealing with them in the process of registration, and the method of filing history and treatment sheets. Add your further remarks.**

**No. 2. (For the Officers of Voluntary Hospitals.)—If you keep your accounts in accordance with the Uniform System, describe fully your method of checking, sorting, analysing, and filing your accounts for payment. Give such particulars as will clearly indicate the manner in which these are passed through your analysis journal, and onwards through the ledger. Add your further remarks.**

RULES.

The following rules must be observed :—

1. Contributions must be written on one side of the paper. They must bear the name and address of the sender and be accompanied by coupon to be cut from the back cover (inside page) of the current issue of The Hospital. A pseudonym must be chosen if the name is not to be published.

2. They must be addressed to the Editor of The Hospital, 28 & 29 Southampton Street, Strand, London, W.C., so as to reach him before the end of the month, and must be marked in the left-hand corner "Facts and Figures."

**A minimum payment of Half a Guinea will be made for each published answer.**

### Laundry Equipment.

The following laundry arrangements at a small provincial isolation hospital are sent us by a correspondent :—The average weekly number of articles washed is 200. There are two tanks for soaking clothes before washing fitted with hot and cold soft-water taps; a boiling tub, boiled by waste steam from engine; a rinsing tub for clothes after boiling, fitted with cold soft-water tap. After soaking the clothes are washed in Bradford's washer, which is fitted up with two cold taps and steam connected to bottom of washer. From the blueing tub the clothes are put into a centrifugal dryer (Bradford's), and from there into a steam-heated drying chamber.

### Needlework Guilds.

There is scarcely a more effective way by which ladies can help an institution than by the formation of a Needlework or Linen Guild. The first step is to secure the services of a popular and influential lady as president, who should be associated with twelve to twenty ladies as a committee, and each of these ladies undertakes to supply a certain number and class of garments each year. They may each have as many associates working for them as they like, who undertake to supply a stated minimum of articles. Frequently the associates meet in turn at the house of one of the committee ladies when the cutting-out, etc., is done; but this is not a necessity, providing the members of the committee draw up a definite scheme of work and give clear directions as to the class and sizes of garments required, the materials to use, etc. A number of hospitals, both large and small, have their Needlework Guild and it forms a most useful adjunct to a crèche and numerous other institutions. The Needlework Guild committee should meet at the institution at least once a year, when a report should be submitted as to the number of articles supplied, etc. The meeting, if held in the summer, may take the form of a garden party, the expenses of which are defrayed by a small subscription (a few shillings) from each committee lady. Such a gathering gives a pleasant opportunity for the associates to meet together, when, in addition to a report as to the working of the Guild, one or two short effective speeches may be made describing the work of the institution generally.

### THE EDITOR'S LETTER-BOX.

### *THE SICK AND IN NEED.*

### Approved Home.

We have pleasure in adding the following Home to our Approved List :—

Norfolk.—Bramblewood, Holt. Principal : Miss M. F. Rumball, certificated nurse. Specially suitable for tuberculous cases. Open-air shelters provided in grounds two acres in extent, situated among pine-woods. Terms, including medical attendance, £3 3s. weekly for adults, and £2 2s. for children.

### Home Offered.

An opportunity occurs at a Home in Surrey for phthisical children (early cases) for a nurse, who herself may be phthisical, to find a home and employment. A trained or partly trained nurse, who has been phthisical but is quite fit for ordinary work and who would be glad to work under open-air conditions and among children would be suitable. Board, lodging, laundry, and a salary would be given to such a nurse. In the alternative a nurse would be received who is not fit for ordinary work but yet could manage, say, five or six hours daily; she would receive board, lodging, laundry, medical attendance, and a certain amount of care and treatment, but no salary. She must, of course, not be an advanced or a bad hæmorrhage case. There are two probationers, both slightly phthisical, whose work the nurse would be required to supervise, and also to do the usual work among the children; but as only early cases are taken there is no heavy nursing. This opportunity may appeal to a nurse requiring an open-air life who on leaving a sanatorium may find it difficult to get a post.—Replies to "C. P. M.," 168b.

### A Very Urgent Need.

We are glad to be able to report that the case we described last week under this head has been partly relieved by the reception of the breadwinner of the family into a sanatorium, where he is to receive care and treatment which it is hoped may be beneficial to him. He is, however, very ill, and the problem that now remains is what to do to help the wife and two small children. It will be remembered that all they have is the husband's sickness benefit under the National Insurance Act of 10s. per week, and the period for this to continue is limited. One of our readers has very kindly come forward with a suggestion and kind offer of help, and if the wife, who is an ex-elementary teacher, could be given employment, and if meanwhile one or two kindly people would combine together to take charge of this case, the needs of this family might be satisfactorily supplied. The wife lives in the neighbourhood of Tunbridge Wells. Will not some one in that district undertake to interest themselves? Replies to "M. E. F.," 167b.

### *EMPLOYMENT AND TRAINING.*

### Midwifery Postal Courses.

You will find postal courses in midwifery advertised in *The Nursing Mirror* from week to week. We are unable to recommend, but would suggest that as you are intending to enter a hospital for midwifery training you should take their advice as to the desirability of other courses.—E. B.

### English Trained Nurses in Australia, etc.

You will find the subject of nursing in Australia dealt with in *The Nursing Mirror* of February 1, 1913. Canadian nursing is treated in an article in the same journal of February 15, 1913, and for information as to South Africa we refer you to the Bureau columns of The Hospital of June 7, 1913. The Colonial Intelligence League, 36 Tavistock Square, W.C., also give information on this subject, and Miss Philippa Hilsdon, of The Island, Hatzic, British Columbia, who is a trained nurse in residence there, has kindly offered to answer any inquiry addressed to her, which should be accompanied by an international postal coupon (purchasable at a post-office) for the reply.—M. B. C.

### Cottage Resident Work.

Yours is clearly a case for advertisement when you should get all the nurses you need.—E. H.

### Opening for Daily Nursing.

In most districts where the better class working people or the lower-middle class live there is an opening for the trained visiting nurse, but it is desirable that you should first be known, or should make yourself known, to the doctors and clergy in the locality. After settling on a neighbourhood it would be well for you to take rooms for a while, to see how the work comes in; later on if you found your services in demand you could take a house, but you must not expect the work to be self-supporting for at least two years. The usual fee is from 1s. a visit in chronic cases and 1s. 6d. for helping at minor operations.—"Nancy."

---

**The Editor is prepared to make known without charge the needs of any who may be sick or in difficulties, and to guide them in making choice of Homes for treatment or convalescence. Reduced terms can often be arranged with Homes on the Approved List for those unable to pay full fees. No Home is included in the Approved List without medical and other testimony to its good management. Queries are specially invited from those who are engaged in any kind of philanthropical work. A new edition of the leaflet describing the purposes of the Bureau of Information is now ready and will be sent free of charge on application. For Rules for Correspondents see page 1.**

### *MISCELLANEOUS.*

### Ice Machine.

The "W.K." hand ice-machine, manufactured by Hannefords, Limited, 91-3 Queen Victoria Street, London, E.C., makes ice in a few minutes, and is suitable for use in any climate. The machine is quite simple and weighs only 50 lb., its size being 18 by 21 by 15 inches.—"Kitty."

### Pamphlet Wanted.

The title of the article you refer to is "Mucous Colitis : its Relationship to Appendicitis and Pericolitis, and its Treatment by Irrigation," by Dr. A. Mantle, 62 York Place, Harrogate, which appeared in the *British Medical Journal* in 1908. It is not published in pamphlet form.—M. P.

### Staff for a Cottage Hospital.

In a small hospital of, say, five beds, where most of the patients are chronic or sub-acute cases, it is usual for the matron to do all the nursing without a probationer, providing she can get efficient assistance when it may be required.—"Matron."

---

### *ACKNOWLEDGMENTS.*

**C. P. M.**—Thanks for letter and photographs. We are very glad to know the Bureau has been helpful to you. We are publishing the information you send and will forward any replies.

**"Nurse."**—We have your card and note you will deal with the matter after you have left the urgent case you are now with.

**P. B. B.** (U.S.A.).—We are very glad to have been able to send you the document you required, and shall be glad at any time to be of service. It is pleasant to know that correspondents in America find the Bureau of use. There is nothing to reimburse to us, as we make no charge for our services.

**M. E. F.**—Thanks for your letter. We are glad to know the case has now been received into a sanatorium. We have forwarded you a letter containing a suggestion in regard to the wife, and are appealing again on her behalf. What would she do with the children if she managed to get employment?

**J. W.**—We are grateful for your kind letter. It is very encouraging to know that you find The Hospital "such a capital paper," and that the Bureau is "of great service to all." We have sent forward your kind offer of help to the case you are interesting yourself in, and have handed on the suggestion contained in the postscript of your letter to the Editor.

**Sister A.**—Thanks for your letter and enclosure, which have been dealt with by the Manager. As to your inquiry, see the answer to M. B. C. in these columns this week, and read the articles referred to which will give you the information you need. You could also get further particulars from the quarters indicated.

### Editor's Notices.

**Contributions :**

**Contributions should be written, or preferably typed, on one side of the paper only, and all articles sent in are accepted upon the distinct understanding that they are forwarded to THE HOSPITAL only.**

**The Editor cannot undertake to return MSS. not used, but when a stamped directed envelope is enclosed the MSS. may be returned if a special request is made.**

**Accepted articles and paragraphs of news will be paid for after publication at the scale rate.**

**Address :**

To prevent delay all contributions and letters on editorial business must be addressed exclusively to the Editor, "The Hospital," 29 Southampton Street, Strand, London, W.C. It is important that this regulation shall be strictly observed.

**Correspondence :**

Correspondence on all subjects is invited. The name and address of correspondents must be given as a guarantee of good faith, but not necessarily for publication.

**Special Articles :**

Special articles are invited, and questions, inquiries, and paragraphs upon all matters relating to the work, administration and management of general, special, mental, fever, cottage and convalescent hospitals, sanatoria, homes, institutions, societies and organisations for the treatment or care of the sick, injured and dependants of all classes. Every matter affecting the interests and welfare of the staff of all grades working in these institutions will receive special consideration.

**A Bureau of Information :**

**We invite inquiries and applications for information and help in providing for that numerous class of sufferers who require special care or house-room which they cannot obtain in their own homes or secure for themselves. We cannot, however, prescribe, or recommend practitioners.**

**Books for Review :**

**Publishers are particularly requested to send advance proofs of any new books of importance whenever possible, as the Editor has made arrangements to publish immediate reviews on a new plan.**

### To Secure an Appointment.

If you are desirous of securing a post in any department of the International, Health, and Sanitary services, The Hospital affords an exclusive and successful medium of direct approach to the Governing Bodies and Executive Officials of Hospitals, Poor-Law Infirmaries, Asylums, and Institutions of every description throughout the United Kingdom, in addition to those responsible for the Administration of Public and Municipal affairs.

It is an indisputable fact that in seeking an appointment success is largely dependent upon the choice of a suitable channel for giving adequate publicity to your capabilities and requirements.

"The Institutional Worker" Supplement is especially designed to enable workers to bring their needs prominently to the notice of those in authority, and a column is reserved for announcements of "Appointments Wanted," in which an advertisement of 21 words can be inserted for the nominal charge of 1s.

If you are seeking re-engagement or desire a change you should at once avail yourself of the exceptional facilities which "The Institutional Worker" provides. It has repeatedly proved itself a valuable help to others in similar circumstances, and you would be well advised to employ *it always* whenever the occasion arises.

### Manager's Notices.

**Letters relating to the publication, sale, and advertising departments of "The Hospital" must be addressed to The Manager, "The Hospital," The Hospital Building, 28 and 29 Southampton Street, London, W.C.**

**Advertisements:**

To ensure insertion, all advertisements for The Hospital must reach the Manager not later than Wednesday morning in each week. For scale of charges see pages v and 4.

**Subscriptions** may begin at any time, and are payable in advance. Cheques and Post-Office Orders should be crossed London County and Westminster Bank, Covent Garden Branch, and made payable to the Manager of The Hospital.

Rates of Subscription (payable in advance).

| | | |
|---|---|---|
| UNITED KINGDOM. | | |
| Three Months (including Postage) | ... | 2s. 0d. |
| Six Months | do. ... | 4s. 0d. |
| Twelve Months | do. ... | 6s. 6d. |
| FOREIGN AND COLONIAL. | | |
| Three Months (including Postage) | ... | 2s. 6d. |
| Six Months | do. ... | 5s. 0d. |
| Twelve Months | do. ... | 8s. 8d. |

**Remittances:**

**It is especially requested that remittances be made by Cheque or Postal Order, and in halfpenny stamps only when the amount is under sixpence.**

*NOW READY.* "The ideal of what a work of reference ought to be."—*The Times.*

**Price 10s. 6d. net; 10s. 11d. Post Free.**

### Burdett's Hospitals and Charities, 1913

THE YEAR BOOK OF PHILANTHROPY, :: AND HOSPITAL ANNUAL ::

*The 1913 Edition contains special Chapters on:*

**The Volume of Charity.**
**The King's Fund and the League of Mercy.**
**British Hospitals and National Insurance.**
**Hospital Construction, 1912.**
**The Nursing Department and its Cost.**
**Hospital Sunday. Hospital Saturday.**

**General Charities—**
**Home and Foreign Missions.**
**Orphanages, Homes and Refuges.**
**Hospital Finance—**
**Income in 1911.**
**Expenditure in 1912.**
**The United States, Canada, Australasia and India.**

"In its own sphere 'Burdett's Hospitals and Charities' has become as well known and as valuable an annual as the other 'Burdett' has long been in the world of finance. The new edition contains, as usual, a mass of carefully compiled information relating to hospitals and charities, and in addition a series of suggestive chapters on general questions in regard to the finances and management of such institutions, particularly the voluntary hospitals in which Sir Henry Burdett is so keenly interested."—*Truth.*

***IMPORTANT.***—Hospital Secretaries and those who require a copy of this Standard Work of Reference should place their order at once either with their Booksellers or with the Publishers, as the Edition is limited.

**THE SCIENTIFIC PRESS, LTD.,** 28 & 29 SOUTHAMPTON ST., STRAND, LONDON, W.C.

### HANDBOOKS
### For TRAINED WORKERS

POCKET·GUIDE S.P. SERIES

**1/- net.** **1/1 Post free.**

**Bound in Limp Cloth.**
**Suitable Size for the Pocket.**

The works which comprise the S.P. Pocket Guide Series are intended for ready reference, consequently the aim of the Publishers has been to make them as concise and lucid as possible. Each book is written by an expert on the subject of which it treats, and the utmost reliance therefore can be placed in the information it imparts.

**Essentials of Fever Nursing.** By LYTTON MAITLAND, M.D. (Lond.), M.B., B.S., D.P.H. (Camb.).

**Manual and Atlas of Swedish Exercises.** (*With over 60 Illustrations.*) By THOMAS D. LUKE, M.D., F.R.C.S.

**Bandaging Made Easy.** (*Illustrated with over 90 Diagrams.*) By M. HOSKING, Sister-in-Charge, Tredegar House, Bow, E.

**How to Write and Read Prescriptions.** By LYTTON MAITLAND, M.D. (Lond.), M.B., B.S., D.P.H. (Camb.).

**Principal Drugs and their Uses.** By A PHARMACIST.

**Asepsis and How to Secure It.** By H. W. CARSON, F.R.C.S.

**Treatment after Operations.** *Rules for Nursing after General and Special Operations.* By MARY WILES.

**The Nurse's Duties before and during Operations.** By MARGARET FOX, Matron, Prince of Wales's General Hospital, Tottenham.

**Other works, in amplification of this Series, will be announced in due course.**

*Published by*

**The SCIENTIFIC PRESS, Ltd.**

**28/29 Southampton Street, STRAND, LONDON, W.C.**

